# Over-Expression of a Tobacco Nitrate Reductase Gene in Wheat (*Triticum aestivum* L.) Increases Seed Protein Content and Weight without Augmenting Nitrogen Supplying

**DOI:** 10.1371/journal.pone.0074678

**Published:** 2013-09-09

**Authors:** Xiao-Qiang Zhao, Xuan-Li Nie, Xing-Guo Xiao

**Affiliations:** State Key Laboratory of Plant Physiology and Biochemistry, College of Biological Sciences, China Agricultural University, Beijing, China; Purdue University, United States of America

## Abstract

Heavy nitrogen (N) application to gain higher yield of wheat (*Triticum aestivum* L.) resulted in increased production cost and environment pollution. How to diminish the N supply without losing yield and/or quality remains a challenge. To meet the challenge, we integrated and expressed a tobacco nitrate reductase gene (*NR*) in transgenic wheat. The 35S-NR gene was transferred into two winter cultivars, “Nongda146” and “Jimai6358”, by *Agrobacterium*-mediation. Over-expression of the transgene remarkably enhanced T_1_ foliar NR activity and significantly augmented T_2_ seed protein content and 1000-grain weight in 63.8% and 68.1% of T_1_ offspring (total 67 individuals analyzed), respectively. Our results suggest that constitutive expression of foreign nitrate reductase gene(s) in wheat might improve nitrogen use efficiency and thus make it possible to increase seed protein content and weight without augmenting N supplying.

## Introduction

Wheat (*Triticum aestivum* L.) is one of the most widely cultivated and most important food crops in the world, and its higher yield depends on heavy field-supply of nitrogen (N) fertilizer [1]–[3]. However, the N use efficiency of crops was low (approximately 33%) [4], [5] and over 50% of the N applied was lost from the plant-soil system [6], leading to environmental damage and negative impacts on human health [7]–[10]. That was particularly pronounced in the areas along the Yellow River, Huai Rover and Hai River (called “Huanghuaihai Area”) in central China [11] where is one of the major areas of wheat production but with saline and alkaline sandy soils and relatively lower yield.

Nitrate (NO_3_^−^) is the main N source for crops under normal field conditions [9], [12], [13] and its availability strongly affects crop productivity and food quality [14], especially in wheat [15]–[17]. The nitrate up-taken in plant is well known to be first reduced to nitrite and then to ammonium via the Glutamate synthesis cycle (GOGAT cycle) in two successive steps catalyzed by nitrate reductase (EC 1.6.6.1, NR) and nitrite reductase (EC 1.7.7.1, NiR) in cytosol and chloroplast, respectively [18]. Thus, the NR is considered a key enzyme in the overall process of nitrate assimilation [19], and how to increase NR content and/or activity, therefore, becomes one of the major challenges for increasing N use efficiency in crops including wheat. Using biotechnology to introduce and over-express exogenous tobacco NR gene was tested for lowering nitrate content in the leaf and edible organs of dicotyledonous crops [20]–[27], but no information about the effect on seed protein content and grain weight was released. To the best of our knowledge, integration and over-expression of foreign NR gene have not been tested in wheat although its foliar NR activity was demonstrated significantly correlated with yield [17], [28], flour quality [16] and grain protein content [15], [17]. The purpose of the present work was to test whether or not introduction and expression of a foreign NR gene in wheat could increase N use efficiency and hence improve quality and/or yield without augmenting N supply, or could maintain quality and/or yield with a diminished use of N fertilizer. Our results demonstrated that over-expression of a CaMV 35S-driven NR gene in two cultivated winter wheat cultivars remarkably enhanced foliar NR activity and significantly increased seed protein content and grain weight under normal soil N conditions.

## Materials and Methods

### Explants and *Agrobacterium tumefaciens-*mediated transformation

Two winter wheat (*Triticum aestivum* L) cultivars, “Nongda146” (ND146) and “Jimai6358” (JM 6358) which are widely cultivated in the “Huanghuaihai Area”, China, were used throughout this study. Their immature embryos were isolated from the young caryopses 12–14 days after anthesis, and induced to produce embryogenic callus as previously described [29]. The calli were pretreated for 8–12 h on an osmotic medium with 0.4 M mannitol before *Agrobacterium tumefaciens* (strain LBA4404) inoculation. The LBA4404 harbored a binary vector pBCSL16 [21] which was kindly provided by Drs. Cabouche and Meyer (INRA, France). The vector carried a kanamycin-resistant gene (*Npt II*) and tobacco nitrate reductase cDNA (*nia*) which was functionally fused to CaMV 35S promoter and terminator. The inoculation and co-culture of the pretreated calli with *Agrobacterium* were performed as previously reported [29].

### Selection and regeneration of G418-resistant wheat plants

After co-culture, the calli were subcultured, G418-resistance selected and shoot-regenerated, and the regenerated green shoots rooted as previously described [29] except that G418 (Geneticin, an aminoglycoside antibiotic similar in structure to gentamicin B1; 25 mg/L) instead of PPT was used as the selective agent. The plantlets were vernalized for 2 weeks at 4°C, and then transplanted in pots in greenhouse and self-fertilized to produce T_1_ seeds. During greenhouse stage, one young leaf from each independent T_0_ transformant and WT was sampled for PCR verification.

### Screening and cultivation of kanamycin-resistant T_1_ plants

Screening of kanamycin-resistant (Kan-R) T_1_ plants was conducted according to Xi and co-workers [30] and Zhang et al. [31] with slight modification. Briefly, the Kan tolerant threshold of WT (ND146 and JM6358) was first determined. The seeds were germinated in a set of Kan concentration (0, 40, 60, 80, 100, 120, 160 or 200 mg/L) at room temperature, and the seedlings were transferred into vermiculite-containing Petri dishes, irrigated with corresponding concentration of Kan and vernalized for 2 weeks at 4°C. After vernalization, the cultures were irrigated with water and placed under the conditions of 25±1°C and 16/8 h (light/dark) photoperiod of ca. 3000 lux. About one week later, green and white seedlings were accounted for each Kan concentration, and the lowest Kan concentration that resulted in more than 90% white seedlings was chosen as the threshold. In order to select T_1_ transformant, the T_1_ seeds were germinated and seedlings were selected as WT except with Kan at the threshold concentration. The green seedling was considered Kan-R.

The Kan-R T_1_ plants were further verified by PCR, and then transplanted in flowerpots (14×16.5 cm) together with untransformed control (WT) in greenhouse, one plant per pot. All pots contained equal quantity of the nutrient soil (1 vermiculite: 3 garden nutrient soil) and were randomly placed in an experimental plot with normal field managements.

### PCR analysis

Total genomic DNA was isolated from fresh leaves using CTAB method developed by Doyle [32] with modifications described by Barro et al. [33]. PCR primers for amplification of a 735 bp fragment from *npt II+nos-*ter were 5′-CTGGGCACAACAGACAAT-3′ (forward) and 5′-GAACGATCTCAGAAGAACTCG-3′ (reverse). The PCR reaction mixture of 20 µl was consisted of 2 µl of LaTaq PCR buffer, 1 µl genomic DNA (100 ng/mL), 2 µl dNTP (2.5 mM), 0.5 µl each primer (10 mM), 0.5 µl LaTaq DNA polymerase (5 U) (Tiangen, Tianjin, China) and 13.5 µl sterile distilled water. The PCR was run at the condition: 95°C for 5 min, followed by 35 cycles of 94°C for 30 s, 60°C for 40 s, 72°C for 40 s and 72°C for 10 min. PCR products were visualized by electrophoresis in 0.8% (w/v) agarose gel containing ethidium bromide.

### Southern blot analysis

Southern blot analysis of PCR products was used to verify PCR-positive T_0_ transformants, and both PCR and Southern blot to identify T_1_ progeny.

For Southern blotting of PCR products, the PCR was run as described above with the genomic DNA from PCR-positive T_0_ transformants as template. PCR products were separated by electrophoresis in 0.8% (w/v) agarose gel. For Southern identification of T_1_ progeny, about 30 µg of genomic DNA from T_1_ individuals or the control (WT) were digested at 37°C for 12 h with *Nde* I that has no recognized site in the T-DNA region of pBCSL16. The digested DNA was fractionated in 0.8% (w/v) agarose gel by electrophoresis run at 22 V for approximately 8 h. The PCR DNA and fractionated DNA were then transferred onto positively charged Hybond™-N+ nylon membrane (Amersham Pharmacia Biotech) by capillarity and fixed by UV cross-linking. The membranes were hybridized using the probe of *npt II+nos* fragments that were labeled with digoxigenin using the random primer labeling kit (DIG DNA Labeling and Detection Kit). Pre-hybridization, hybridization and detection of the probe were carried out using a non-radioactive, DIG Luminescent Detection Kit for Nucleic Acids (Roche Diagnostics) according to the manufacturer’s instructions.

### Determination of nitrate reductase activity

The nitrate reductase activity (NRA) was measured *in vivo* according to Freschi et al. [34] with slight modification. Real and potential NRAs were those measured without and with KNO_3_ induction, respectively. The fresh flag leaf at grain filling period was collected between 9:00 and 10:00 a.m. from greenhouse-grown wheat, and cut into equal two parts along the main vein. One part was used for measurement of real NRA and another part, for potential NRA. To measure the potential NRA, the sample was first induced in 50 mM KNO_3_ for 12 h at 25°C under light of 3000 lux, and then vacuum-infiltrated. For vacuum-infiltration, leaf samples (0.2 g fresh weight) with or without KNO_3_ induction were cut into pieces (0.5–1 cm^2^), immersed in an incubation buffer (5 ml phosphate buffer (pH 7.5) + 5 ml 0.2 M KNO_3_ solution), vacuum-infiltrated 3–4 times, each for 20 min, and then incubated in darkness for 30 min at 30°C. After infiltration, the nitrate reduction was carried out at room temperature for 30 min in a reaction mixture containing 1 ml of sample infiltrate, 1 ml of 1% (w/v) sulfanilamide in 36% HCl and 1 ml of 0.2% (w/v) 1-naphthylamine. The nitrite (NO_2_^−^) formed was detected spectrophotometrically at 540 nm, and the NRA was expressed inµg of nitrite (NIR) produced per hour and per gram of fresh leaf. The experiment was triplicated.

### Measurement of nitrate contents

The foliar nitrate content was determined according to Cataldo et al. [35] slightly modified. Leaf segments were dried at 85°C until constant weight. The dried material (25 mg) was grounded to powder and then incubated in 10 ml of distilled water for 2.5 h. Aliquots of 0.1 ml were mixed thoroughly with 0.4 ml of 5% (w/v) salicylic acid in concentrated H_2_SO_4_. After 20 min incubation at room temperature, 9.5 ml of 2 M NaOH were added. The samples were cooled to room temperature and nitrate concentration determined spectrophotometrically by measuring the absorbance at 410 nm.

### Protein and 1000-grain weight analysis of T_2_ seeds

At harvest, the seeds from 57 T_1_-individuals with good seed-setting rate were chosen for determination of 1000-grain weight and protein content. To determine 1000-grain weight, 15 seeds per individual plant were picked up randomly and weighted. For detecting protein content, the seeds were first dried at 40°C to constant weight, and then milled and sieved (100 mesh). The protein content of the flour was blindly measured by a commercial company using Kjeldahl method with a continuous flow analyzer (Auto Analyzer 3 Bran^+^Luebbe, Germany) on three replicates, and calculated by using a conversion factor of 5.7.

### Statistical analysis

Data of NRA, nitrate content, protein content and grain weight were analyzed by analysis of variance (ANOVA) followed by Duncan’s multiple test and *T-test* with SPSS 17.0 software (SPSS Inc, Chicago, IL, USA).

## Results

### Transformation and regeneration of transgenic wheat

**Figure 1:**
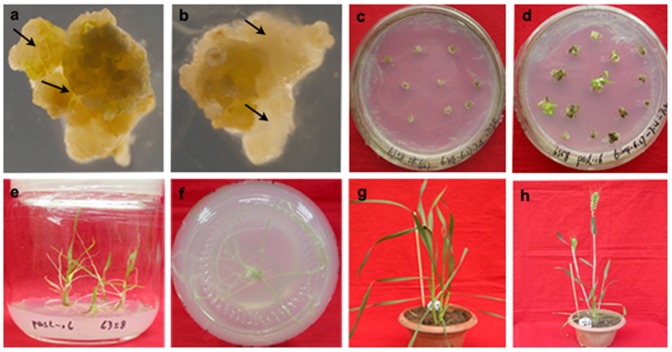
*Agrobacterium*-mediated transformation and regeneration of transgenic plants from immature embryo-derived callus of common wheat (*Triticum aestivum* L.). a: Embryogenic calli (→) formed from immature embryos. b: Non-embryogenic calli (→) formed from immature embryos. c: Untransformed embryogenic calli (Control) on the regeneration medium supplemented with 25 mg/L G418. d: Shoot regeneration from *Agrobacterium*-infected embryogenic calli on the regeneration medium supplemented with 25 mg/L G418. e & f: Rooting of regenerated shoots on the rooting medium supplemented with 25 mg/L G418. g: G418-resistant plant in pot. h: Fertile G418-resistant plants in pot.

Under promoting conditions of callus induction, 91.8% and 96% of immature embryos from JM6358 (2450 embryos cultured) and ND146 (3645 embryos cultured) developed embryogenic and non-embryogenic calli (Fig. 1a & 1b), respectively. After co-culture with the *Agrobacterium* and selected on G418-containing medium, 51.2% (1024/2000) and 86.2% (2843/3300) of embryogenic calli from JM6358 and ND146 formed resistant callus, whereas the calli from WT (not infected with the bacterium) became browning. On the regeneration medium containing G418, the WT calli ceased growing and did not differentiate (Fig. 1c), but the resistant calli regenerated green shoots (Fig. 1d) at the frequency of 42.1% (510/1210) and 58.5% (1650/2820) for JM6358 and ND146, respectively. In G418-containing rooting medium, 17.4% (21/121) and 34% (96/282) of green shoots from JM6358 and ND146 rooted (Fig. 1e & 1f), but no one from WT. The plantlets grew well and were fertile after transplanting in pots in greenhouse (Fig. 1g & 1h).

### PCR and Southern blot identification of T_0_ transformants

**Figure 2:**
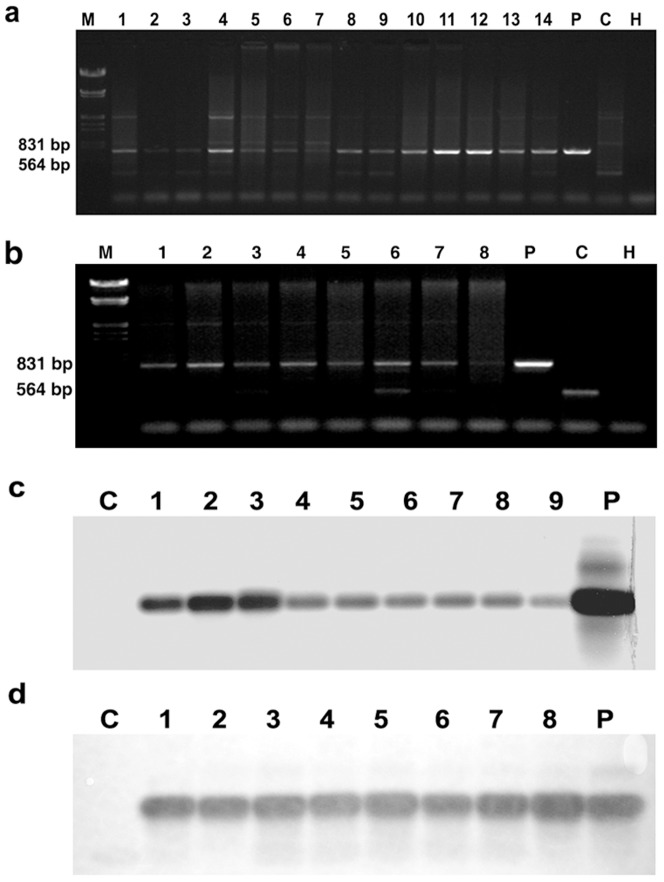
PCR and Southern analysis of PCR products identification of T_0_ transformants of wheat. PCR (**a, b**) and Southern analysis of PCR products(**c, d**) detection of *npt II*+*nos* fragment in G418-resistant T_0_ transformants of ND146 (**a, c**) and JM6358 (**b, d**). In **a** and **b**: M: Molecular weight DNA markers (λDNA/*Eco*R I +*Hin*d III). Lanes 1-14: G418-resistant T_0_ plants from independent transformation events. P: Vector plasmid. C: Control (untransformed plant). H: H_2_O (PCR mix without DNA). The arrow indicates the 735 bp fragment of *npt II*+*nos*. In **c** and **d**: Lanes 1-9: PCR-positive T_0_ plants from independent transformation events. P: Vector plasmid. C: Control (untransformed plant).

Among independent G418-resistant T_0_ transformants, 8 and 53 individuals from JM6358 and ND146 had one expected band of about 740 bp in the PCR product (Parts shown in Figs. 2a & 2b). This gave a transformation efficiency of 0.4% (8/2000) and 1.6% (53/3300), respectively, based on the number of PCR-positive plants/number of the embryogenic calli trans-infected. When Southern blotted, all PCR-positive products and the vector plasmid had a clear hybridized band, whereas no such a band appeared from untransformed control plant (Fig. 2c & 2d).

### Kanamycin screening of T_1_ offspring and PCR verification of the screening

**Figure 3:**
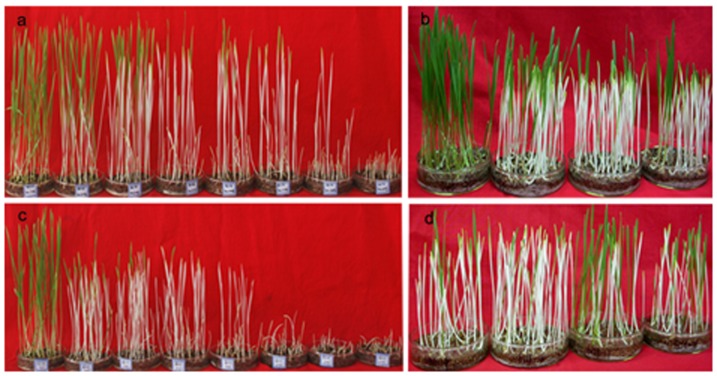
Kanamycin screening of T_1_ offspring of transgenic wheat. Seed germination and seedlings growth of wild-type ND146 (**a**) and JM6358 (**c**) in kanamycin (Kan) solution at different concentrations (0, 40, 60, 80, 100, 120, 160 and 200 mg/L Kan from left to right). WT seedlings are albino at and over 80 mg/L Kan whereas some seedlings from T_1_ seeds of NR-ND146 (**b**) and NR-JM6358 (**d**) remains green in 80 mg/L Kan solution after 2-weeks screening.

In the tested concentrations of Kan solution (0, 40, 60, 80, 100, 120, 160 or 200 mg/L), 95% of the WT seeds germinated, but more than 90% of the seedlings were albino when Kan concentration reached at 80 mg/L or more (Fig. 3a & 3c). In 80 mg/L of Kan solution, 1.4%–89.2% T_1_ seedlings from 9 independent T_0_ lines of NR-ND146 (73-212 plants) and 4.9%–49.4% from 7 T_0_ lines of NR-JM6358 (61–87 plants) remained green (Fig. 3b & 3d).

The T_1_ green seedlings were further verified by PCR. Overall 71.6% and 70.6% T_1_ green seedlings of NR-ND146 (225 plants) and NR-JM6358 (85 plants) were PCR-positive (PCR+), respectively (Table 1), but none of the albino seedlings from two families were PCR+ (Data not shown). As presented in Table 1, in NR-ND146 family 7 out of 9 lines had a ratio of 1 : 1 of the Kan-R : PCR+ individual, whereas in the family NR-JM6358, this ratio was only noted in 1 of 7 lines.

**Table 1:** Kanamycin screening of T_1_ transformants of wheat and PCR verification of the screening.

T_0_ line	No. of T_1_ seeds	No. of Kan-RT_1_ plants	No. of PCR+T_1_ plants	Kan-R:PCR+
NR-ND146-11	69	2	2	1:1
NR-ND146-27	36	1	1	1:1
NR-ND146-49	88	5	5	1:1
NR-ND146-50	94	7	7	1:1
NR-ND146-90	212	189	128	1.48:1
NR-ND146-93	103	7	5	1.4:1
NR-ND146-95	105	9	8	1.13:1
NR-ND146-104	73	1	1	1:1
NR-ND146-137	20	4	4	1:1
**NR-ND146 Total**		**225**	**161**	1.4:1
NR-JM6358-1	57	5	2	2.5:1
NR-JM6358-5	69	5	3	1.67:1
NR-JM6358-11	87	43	32	1.34:1
NR-JM6358-14	61	9	4	2.25:1
NR-JM6358-16	61	3	1	3:1
NR-JM6358-17	82	10	8	1.25:1
NR-JM6358-18	80	10	10	1:1
**NR-JM6358 Total**		**85**	**60**	1.42:1

Kan-R: Kan-Resistant; PCR+: PCR positive.

### Southern blot analysis of T_1_ offspring

**Figure 4:**
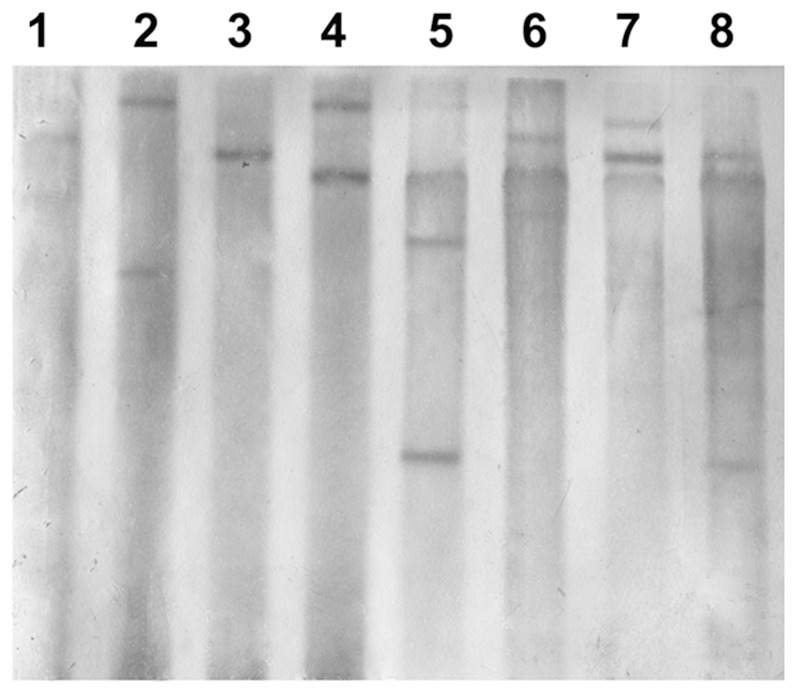
Southern blot analysis of T_1_ transformants of wheat. Lane 1-4: T_1_ offspring of NR-ND146 family (146-50-5, 146-90-87, 146-90-189 and 146-95-4). Lane 5-8: T_1_ offspring of NR-JM6358 family (6358-5-4, 6358-11-20, 6358-11-40 and 6358-17-5).

The presence of the transgene in PCR+ T_1_ progeny was further verified by Southern blot analysis. In 8 PCR+ individuals randomly picked (4 from NR-ND146 and 4 from NR-JM6358), the hybridizing band was clearly present, and the band number varied from 1 to 5, with fewer bands in the individuals from NR-ND146 family (lanes 1-4) than in those from NR-JM6358 (lanes 5-8) (Fig. 4).

### Real and potential NR activities of T_1_ progeny

**Figure 5:**
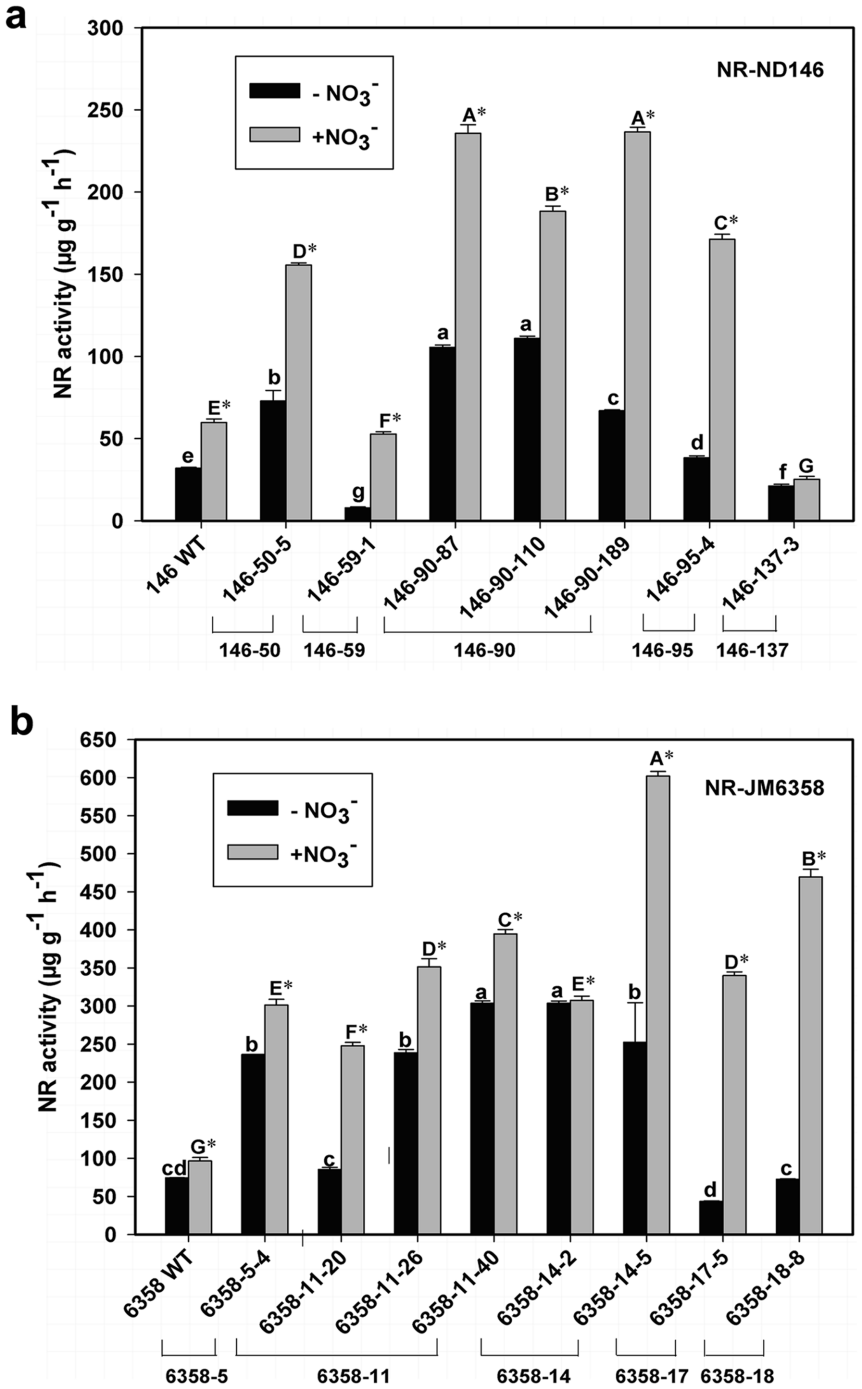
Foliar nitrate reductase activity (NRA) of T_1_ transformants of wheat. The value represents the mean plus SD of three independent experiments, each with three measurements. * denotes significant difference at *P*<0.05 between KNO_3_-induced and non-induced NRAs of the same plant. Different lowercase letters indicate significant difference at *P*<0.05 among individuals without KNO_3_-inducement, and the different capital ones, with KNO_3_-inducement, according to T-test.

The foliar NRA was significantly enhanced by 50 mM KNO_3_ induction, and this increment took place both in WT and T_1_ progeny (Fig. 5). Compared with WT, the T_1_ offspring of NR-ND146 had a significant higher NRA in 5 of 7 individuals tested (146-50-5, 146-90-87, 146-90-110, 146-90-189 and 146-95-4), no matter with or without KNO_3_ inducement (Fig. 5a). However, in NR-JM6358 descendants, all tested T_1_ individuals from 5 T_0_ lines displayed remarkably stronger NRA than WT when induced with KNO_3_. Without the induction, one individual (plant 6358-17-5) even showed lower NRA than WT (Fig. 5b).

### Nitrate content of T_1_ plants

**Figure 6:**
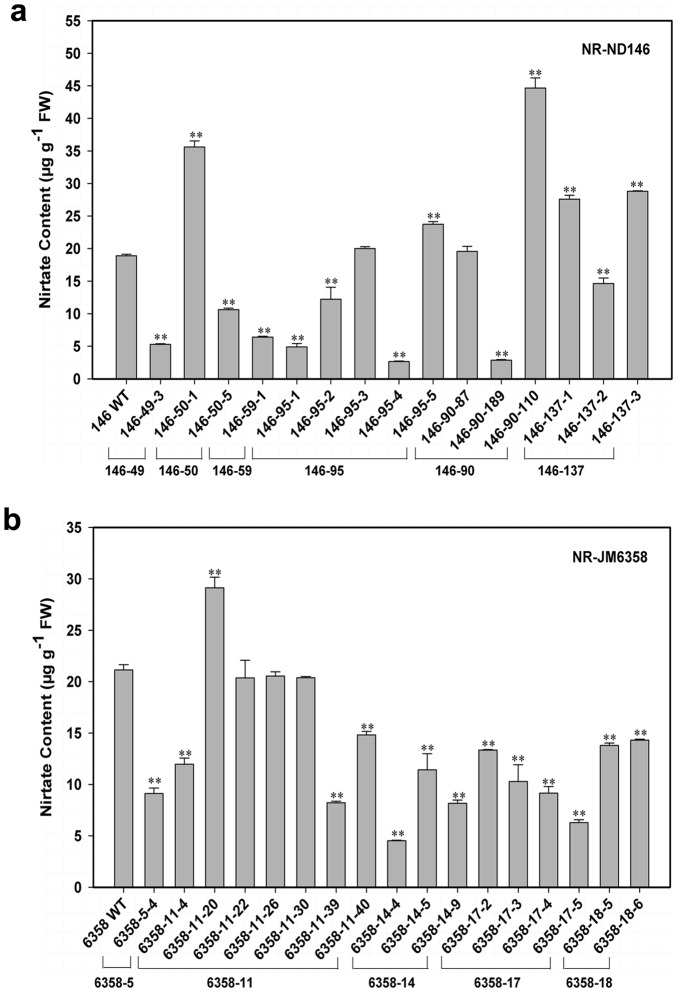
Foliar nitrate content of T_1_ transformants of wheat. Nitrate content in leaves of T_1_ offspring of NR-ND146 (**a**), NR-JM6358 (**b**) and the corresponding WT was determined with and without pre-inducement of KNO_3_. The value represents the mean plus SD of three independent experiments, each with three measurements. ** denotes significant differences at *P*<0.01, according to T-test.

In NR-ND146 family, the foliar nitrate content of 15 T_1_ individuals varied from 2.67 to 44.67 µg/g FW. The lowest, detected in plant 146-95-4, was 14.2% of the WT (18.9 µg/g FW) and the highest, in plant 146-90-110, approximately 2.4-fold of the WT (Fig. 6a). Among 15 T_1_ individuals, 8 plants displayed significantly lower nitrate content than WT, but 5 plants, higher than WT (Fig. 6a).

In the family of NR-JM6358, 13 out of 17 T_1_ descendents had the nitrate content remarkably lower than WT (21.17 µg/g FW), but 1 individual plant, higher than WT (Fig. 6b). The lowest foliar NO_3_^−^ (4.53) was noted in plant 6358-14-4, being 21.4% of the WT, while the highest (29.13), in plant 6358-11-20, about 137.6% of the WT.

### 1000-grain weight and protein content of T_2_ seeds

Mature T_2_ seeds were collected from 27 individuals of 9 T_1_ NR-ND146 lines and 30 individuals of 7 T_1_ NR-JM6358 lines. Among them, 3 individuals from NR-ND146 family and 4 from NR-JM6358 family had the flag leaf sampled for NRA and NO_3_^−^ content determination. In order to exclude the influence of leaf-sampling on seed protein content and grain-weight, we analyzed the T_2_ seeds of the plants whose leaves sampled in one group and the those with intact leaf, in another one.

#### 1000-grain weight

In all leaf-sampled T_1_ plants of NR-ND146 family, the T_2_ seeds had a very significant higher 1000-grain weight than WT, whereas in 4 leaf-sampled T_1_ individuals of NR-JM6358 family, only two displayed such a significant increment, and the rest, much modest (Table 2).

**Table 2:** Protein content and 1000-grain weight of T_2_ seeds.

NR-ND146			NR-JM6358		
T_1_ plants	1000-grain weight (g)	Seed protein content (%)	T_1_ plants	1000-grain weight (g)	Seed protein content (%)
**Leaf-sampled**			**Leaf-sampled**		
ND146 (WT)	28.67±0.58	18.80±0.17	JM6358 (WT)	31.33±1.53	20.31±0.06
146-50-5	42.00±1.00***	25.12±0.12***	6358-5-4	53.33±0.58***	22.69±0.04***
146-90-110	37.67±1.53***	29.12±0.07***	6358-11-20	32.67±0.58	20.24±0.16
146-137-3	not determined	23.05±0.22***	6358-14-2	31.67±1.16	24.62±0.10***
**Leaf-intact**			6358-18-8	36.33±0.58***	23.42±0.08***
ND146 (WT)	33.67±2.08	19.08±0.01	Leaf-intact		
146-11-1	34.33±2.08	27.42±0.16***	JM6358 (WT)	33.33±0.58	21.70±0.02
146-11-2	36.67±1.53	25.65±0.09***	6358-1-2	36.00±1.00*	17.23±0.03***
			6358-1-3	32.67±1.16	17.53±0.15***
146-27-1	45.00±3.61***	23.37±0.09***	6358-1-4	28.33±1.16***	21.32±0.06***
					
146-49-3	48.33±0.58***	20.50±0.07***	6358-5-2	50.00±3.61***	22.88±0.01***
146-49-4	32.67±2.08	27.75±0.05***	6358-5-5	38.00±1.00***	22.55±0.19***
146-49-5	49.67±6.03***	18.73±0.07***			
			6358-11-8	36.33±1.53*	19.02±0.02***
146-50-1	45.33±2.08***	23.83±0.21***	6358-11-12	35.67±0.58*	26.50±0.17***
146-50-2	40.00±1.00***	23.82±0.11***	6358-11-19	38.33±0.58***	22.48±0.02***
146-50-3	45.00±1.00***	24.21±0.121***	6358-11-24	34.67±0.58	21.49±0.06*
			6358-11-29	34.33±1.16	21.94±0.06**
146-90-5	43.00±2.00***	14.351±0.14***			
146-90-10	38.33±1.16**	14.13±0.01***	6358-14-1	32.67±2.08	23.43±0.04***
146-90-27	50.00±1.00***	22.58±0.02***	6358-14-3	33.00±1.73	23.62±0.12***
146-90-109	43.67±1.16***	25.11±0.03**	6358-14-9	47.33±0.58***	22.22±0.02***
146-90-169	26.33±0.58***	22.48±0.05***			
			6358-16-1	32.33±0.58	22.78±0.08***
146-93-2	37.00±1.00*	25.86±0.05***	6358-16-2	33.67±1.16	21.03±0.10***
146-93-3	33.67±1.53	31.49±0.11***	6358-16-3	38.33±1.53***	19.53±0.16**
146-93-5	37.67±1.53*	16.11±0.13***			
146-93-6	42.67±2.08***	23.70±0.06***	6358-17-2	36.67±0.58***	20.36±0.11***
			6358-17-4	43.00±2.00***	22.76±0.04***
146-95-1	38.67±1.53**	28.08±0.02***	6358-17-6	43.33±1.53***	20.13±0.11***
146-95-3	24.33±0.58***	29.17±0.04***	6358-17-7	38.00±1.00***	19.58±0.04***
146-95-6	34.00±1.00	14.83±0.14***	6358-17-9	38.00±1.00***	19.69±0.06***
146-95-7	43.67±1.16***	14.72±0.02***			
			6358-18-1	36.67±1.53**	22.48±0.10***
146-104-1	41.00±1.00***	24.10±0.06***	6358-18-2	39.67±2.08***	18.97±0.06***
146-137-1	40.33±2.31***	16.27±0.08***	6358-18-5	40.00±1.00***	20.17±0.20***
			6358-18-6	34.33±0.58	22.78±0.08***
			6358-18-9	46.33±1.53***	22.96±0.16***

T_1_ plants of 35S-NR-transgenic wheat and wild-type (WT) were randomly grown in greenhouse under conventional conditions. Values represent mean ±S.D. of three replicates. Difference significant at *P*<0.05 (*),<0.01 (**) or <0.001 (***) according to T-test.

In leaf-intact T_1_ plants, 70.8% (17/24) and 65.4% (17/26) of individuals in NR-ND146 and NR-JM6358 families had the grain remarkably weightier than WT, respectively (Table 2). As showed in Table 2, in NR-ND146 family, 45.8% of T_1_ offspring augmented their grain weight by more than 20%, and 20.8% of the individuals, by 10%-20%, compared with WT, whereas in NR-JM6358 family, the same augmentation rate was only found in 19.2% and 34.6% of T_1_ descendants, respectively.

#### Protein content

All leaf-sampled T_1_ plants in both NR-ND146 and NR-JM6358 families had the seed crude protein content much higher than WT (Table 2), and the highest reached at 29.1% in NR-ND146 family (plant 146-90-110) and 24.6% in NR-JM6358 (plant 6358-14-2), being 54.9% and 21.2% higher than their WT, respectively.

Among 24 leaf-intact T_1_ plants of NR-ND146 family, 70.8% increased their seed protein content in comparison with WT (protein content: 19.08%), with an increment range of more than 30% in 33.3% individuals and 20%-30% in 25% individuals. The highest seeds protein content reached at 31.49% (plant 146-93-3) which is 1.65 times of WT.

In NR-JM6358 family, 4 out of 26 leaf-intact T_1_ plants had the protein content higher than the WT (21.7%) by over 5%, 8 T_1_ individuals by 2%-5%, but about one half of individuals even declined their seed protein content, more or less (Table 2).

## Discussion

### Transformation and regeneration of cultivated winter wheat and rapid screening of T_1_ transformants

Although the first report on successful transformation and regeneration of wheat mediated by *Agrobacterium tumefaciens* was reported in 1997 by Cheng et al. [36], most reported transformation events were still limited to some “model” spring-type cultivars such as “Bobwhite” and “Chinese Spring” [37], [38]. We successfully transferred a tobacco nitrate reductase gene (*Nia2*) into two commercially cultivated winter wheat cultivars, “ND146” and ”JM6358” with *Agrobacterium*-mediation and obtained numbers of fertile transgenic plants (Figs. 1 & 2) following our protocol established [39] and improved [29], [40], [41]. We realized a transformation efficiency of 1.68% in “ND146” and 0.40% in “JM6358” based on the number of PCR-positive plants/number of calli inoculated.

After successful transformation and regeneration, we turned our attention to how to select transformants rapidly, efficiently and cost-effectively. In wheat as in other cereals, using hygromycin resistance gene was considered an effective selection system that allowed few escape plants to survive [42]. However, taking consideration of the existing biosafety/regulatory rules about genetically modified crops (GMC) and possible commercial cultivation of the transgenic wheat, we used Kan-R gene (*npt II*) in place of hygromycin-R one as the selection gene. We used G418 in the place of Kan as the selective agent at different *in vitro* stages of the transformation due to wheat’s native resistance to Kan. Our results demonstrated that G418 at 25 mg/L was efficient for selecting *npt II*-transgenic calli, shoots and plantlets at corresponding stages of the transformation (Fig. 1c to 1h). Even so, we were aware that the G418 was much expensive than Kan, and its amount requested for “field” selection of T_1_ and then-after offspring would be much more than the selection *in vitro* of T_0_ transformants. In order to reduce the selection cost, we adopted the method of Xi and colleagues [30] and Zhang et al. [31] by germinating T_1_ seeds in Kan solution in dishes and then choosing green seedlings. Our results showed that more than 70% of the green seedlings in both genotypes (161/225 in NR-ND146 and 60/85 in NR-JM6358) developed in 80 mg/L of Kan solution were PCR-positive (Table 1), and all albino seedlings was PCR-negative (data not shown). The similar results were reported by Zhang et al. [31] and Ren et al. [43] in other genotypes of transgenic wheat. This indicated that *npt II* could be used as selectable marker gene in wheat transformation and Kan was efficient and cost-effective to screen primarily the T_1_ offspring and then-after generations.

### Nitrate reductase activity and nitrate content in the flag leaf of T_1_ progeny

In untransformed wheat, the nitrate reductase activities (NRA) of the leaf tissues [15], [16], basipetal part of the youngest ligule emergent leaf [28], third leaf [44], flag leaf [17] and even shoots [45] were found to be correlated more or less with yield and/or grain (flour) quality. We used the flag leaf for determining NRA and nitrate content of T_1_ progeny, because its NRA was significantly correlated with both yield and grain protein content in winter wheat [17].

Over-expression of 35S-NR gene remarkably enhanced foliar NRA in more than 70% of T_1_ descendants analyzed in NR-ND146 and NR-JM6358 families, and a maximum increment level reached at 3.46 times and 4.08 times of the WT, respectively (Fig. 5). This kind of NRA-increment was also reported in 35S-NR-transgenic dicotyledonous crops, such as in tobacco [20], Arabidopsis [46], lettuce [21], Chinese cabbage (*Brassica. campestris* L. ssp. *pekinensis*) and pakchoi (*B. campestris* L. ssp. *chinensis*) [22]. Our data showed that without NO_3_^−^ inducement, the NRA of T_1_ progeny was T_0_ parent line-dependent, and the different T_1_ individuals from one single T_0_ line had also remarkably varied NRA (Fig. 5). Under NO_3_^−^ inducement, both WT and T_1_ plants enhanced their leaf NRA, but the increment was much more pronounced in T_1_ plants than in WT (Fig. 5), with a maximal 3.9-fold and 6.2-fold increment in the T_1_ offspring of the family NR-ND146 (Fig. 5a) and NR-JM6538 (Fig. 5b), respectively. This implied that both endogenous and transgenic NR genes were nitrate-inducible, at least, in wheat, although the transgene *NR* was driven by constitutive promoter 35S.

Over-expression of 35S-NR gene significantly declined leaf nitrate content in 53.3% (8/15) to 76.5% (13/17) of T_1_ individuals of NR-ND146 and NR-JM6358 families, respectively, with a maximal decrement of 78.6% (plant 6358-14-4) to 85.9% (plant 146-95-4) (Fig. 6). Such decrement of foliar nitrate content was also observed in numbers of NR-transgenic dicotyledonous crops: such as in tobacco [20], [47]–[49], lettuce [21], potato [23]–[25], Chinese cabbage and pakchoi [22], [27]. It was well known that the NR, as a rate-limiting enzyme, catalyzed reduction of NO_3_^−^ into NO_2_^−^, and thus logically over-expression of *NR* could decrease nitrate content in plant. Hu et al. [50] even speculated that the higher NRA was the more nitrate would be reduced. However, in our NR-transgenic wheat under the greenhouse growth conditions, the increment of foliar NRA was sometimes correlated with the nitrate decrement in the leaves of T_1_ offspring of both NR-ND146 and NR-JM6358 families (Fig. S1). Sun et al. [51] reported that Arabidopsis plants transformed with a Chinese cabbage NR gene exhibited an enhanced level of both NO_3_^−^ and NRA in leaves under NO_3_^−^ inducement. Hoff et al. [52] also observed that Arabidopsis mutants affecting *Nia2* and barley *Nar1* mutants expressing only 10% of the WT NRA did not alter nitrate content and biomass under the greenhouse growth conditions. What is the reason remained to be investigated. In our transgenic wheat, the accumulated nitrate in the leaf would be used later for grain development.

### Seed weight, protein content and their relationship with foliar nitrate reductase activity

Our data demonstrated that 70.8% (17/24) NR-ND146 and 50% (13/26) NR-JM6358 T_1_ descendants had significant higher protein content than WT, and a more than 30% increment was detected in 33% of T_1_ offspring in NR-ND146 family (Table 2). For a limited number of leaf-sampled T_1_ plants, the seed protein content looked like have a tendency of positive correlation with foliar NRA in both families (Fig. S2). In non-transformed spring wheat [15] and winter one [17], the foliar NRA was observed positively correlated with seed protein content. Kumari [17] thought that grain protein accumulation depended on the accumulation and partitioning of the reduced N accumulated during the vegetative stage and on the relative contributions of nitrate assimilation and N redistribution during grain development. In N-deficient wheat plants, lower shoot NRA resulted in decrement of reduced N accumulation daily in the shoots [45]. The plants grown in nitrate-rich conditions not only enhanced the activities of NR, ribulose bisphosphate carboxylase-oxygenase (RuBPCO) and glutamine synthetase etc. in growing and full expensed leaves, but also slowed the decrease of those activities in older leaves and delayed leaf senescence [44]. An increase in the supply of glutamine could enhance the rate of protein deposition in the wheat grain [53]. In 35S-NR-transgenic tobacco plants with higher foliar extractable NRA, Ferrario-Méry et al. [54] observed the increased glutamine level in the leaves. We suggested that the increased foliar NRA in 35S-NR-transgenic wheat might speed up nitrate assimilation and facilitate the N-flux to and/or N redistribution in seeds during grain development, and hence increased grain protein content.

We noted that in T_1_ plant with intact leaf, 70.8% and 65.4% of them remarkably augmented their grain weight in NR-ND146 and NR-JM6358 families, respectively (Table 2). In order to know whether the increase in grain weight has some relationship with foliar NRA, we analyzed T_1_ plants whose flag leaves were sampled for NRA and NO_3_^−^ determination. Our data showed that the correlation between grain weight and flag leaf NRA was varied with transgenic wheat families: R^2^ = 1 in NR-JM6538, and R^2^ = 0.4569 in NR-ND146 (Fig. S3). In untransformed wheat, the NRA in the basipetal part of the youngest ligule emergent leaf [28], flag leaf [17] and leaf tissues at boot stage of maturity [15] correlated well with yield. Kumari [17] observed that on induction of NRA by nitrate supply at post anthesis stage, the flag leaf retained the ability to synthesize RuBPCO. We suggested that constant expression of the NR gene in 35S-NR-transgenic wheat might help consistent synthesis of RuBPCO, and thus confer to the leaves longer and higher capacity of photosynthesis, and hence increase grain weight.

**Figure 7:**
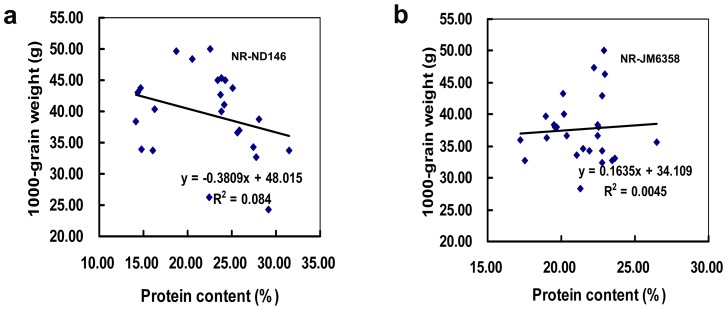
Relationship between T_2_ seed weight and seed protein content of T_1_ wheat progeny with intact leaves. a: NR-ND146. b: NR-JM6358.

Our data indicated also that in 35S-NR-transgenic wheat, there was not obvious relationship between grain weight and seed protein content (Fig. 7), and in some T_1_ individuals, the increment of grain weight was accompanied by the increase of seed protein content (Table 2). Jenner et al. [55] showed that the duration and rate of both starch and protein deposition in the endosperm of wheat were all independent events, controlled by separate mechanisms. Under N-rich growing conditions, both the duration and rate of starch deposition during grain filling were determined primarily by factors that worked close to or within the grain itself, whereas those of protein deposition were decided predominantly by factors of supply outside the grain. In studying the relationships between carbon and nitrogen metabolism in the leaves of NR-transgenic tobacco that expressed either a 5-fold increase or a 20-fold decrease in NRA, Foyer and colleagues [56] concluded that large decreases in NRA had profound repercussions for photosynthesis and carbon partitioning within the leaf, but the increases in NRA had negligible effects. In Arabidopsis, over-expression of *NR* led to 200% increase of seedlings protein content without any gain in the fresh and dry weights [46]. We are aware that more works are needed to address the mechanism of the cell- and organ-specific expression and metabolic regulation of NR gene and other genes involved in the nitrogen assimilatory pathway and to investigate the role of the enzymes in regulating flux through the nitrogen assimilation pathways, as indicated by Cullimore and Bennett [57].

We noted also, there was obvious variability in both seed protein content and grain weight among independent transformants and their progeny (Table 2). Random insertion of the transgene in the genome of T_0_ transformants and random recombination of the transgene in producing the progeny might be one of the explanations, because the insertion might change the expression of adjacent genes [58].

In conclusion, over-expression of 35S-NR gene in winter wheat significantly increased grain weight and seed protein content. This might be realized by an increased foliar NRA. The enhanced NRA might speed up nitrate assimilation and facilitate N-flux to and/or N redistribution in seeds during grain development in one hand, and make the leaf to have longer and higher capacity of photosynthesis, in other hands. Our results would provide an alternative way to breeding new wheat cultivars of higher protein content and higher nitrogen use efficiency, which makes it possible to reduce the need for excessive input of N fertilizers and improve or stabilize quality.

## Supporting Information

Figure S1 **Relationship between foliar NRA and nitrate content of T_1_ transformants of wheat. a**: Leaf-sampled T_1_ offspring of NR-ND146. **b**: Leaf-sampled T_1_ offspring of NR-JM6358.
(TIF)

Figure S2 **Relationship between T_1_ foliar NRA and T_2_ seed protein content of transgenic wheat. a**: Leaf-sampled T_1_ offspring of NR-ND146. **b**: Leaf-sampled T_1_ offspring of NR-JM6358.
(TIF)

Figure S3 **Relationship between T_1_ foliar NRA and T_2_ seed weight of transgenic wheat. a**: Leaf-sampled T_1_ offspring of NR-ND146. **b**: Leaf-sampled T_1_ offspring of NR-JM6358.
(TIF)
