# Supplementary figures and images for: Over-Expression of a Tobacco Nitrate Reductase Gene in Wheat (Triticum aestivum L.) Increases Seed Protein Content and Weight without Augmenting Nitrogen Supplying
Source: PLoS One. 2013 Sep 9;8(9):e74678. doi: 10.1371/journal.pone.0074678 (PMC3767627; doi:10.1371/journal.pone.0074678)

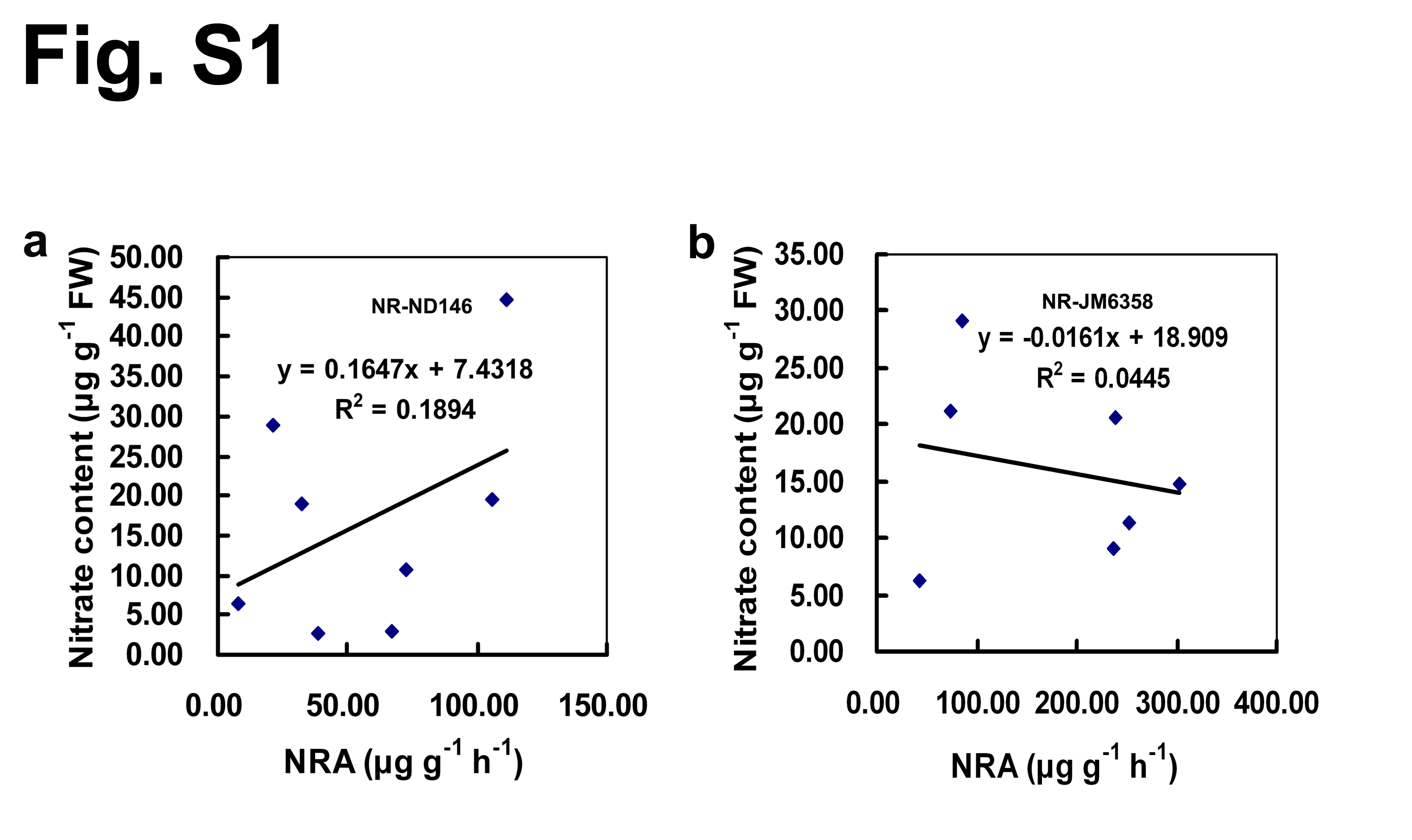

Supplement: Figure S1 — Relationship between foliar NRA and nitrate content of T1 transformants of wheat. a: Leaf-sampled T1 offspring of NR-ND146. b: Leaf-sampled T1 offspring of NR-JM6358. (TIF) [file pone.0074678.s001.tif]

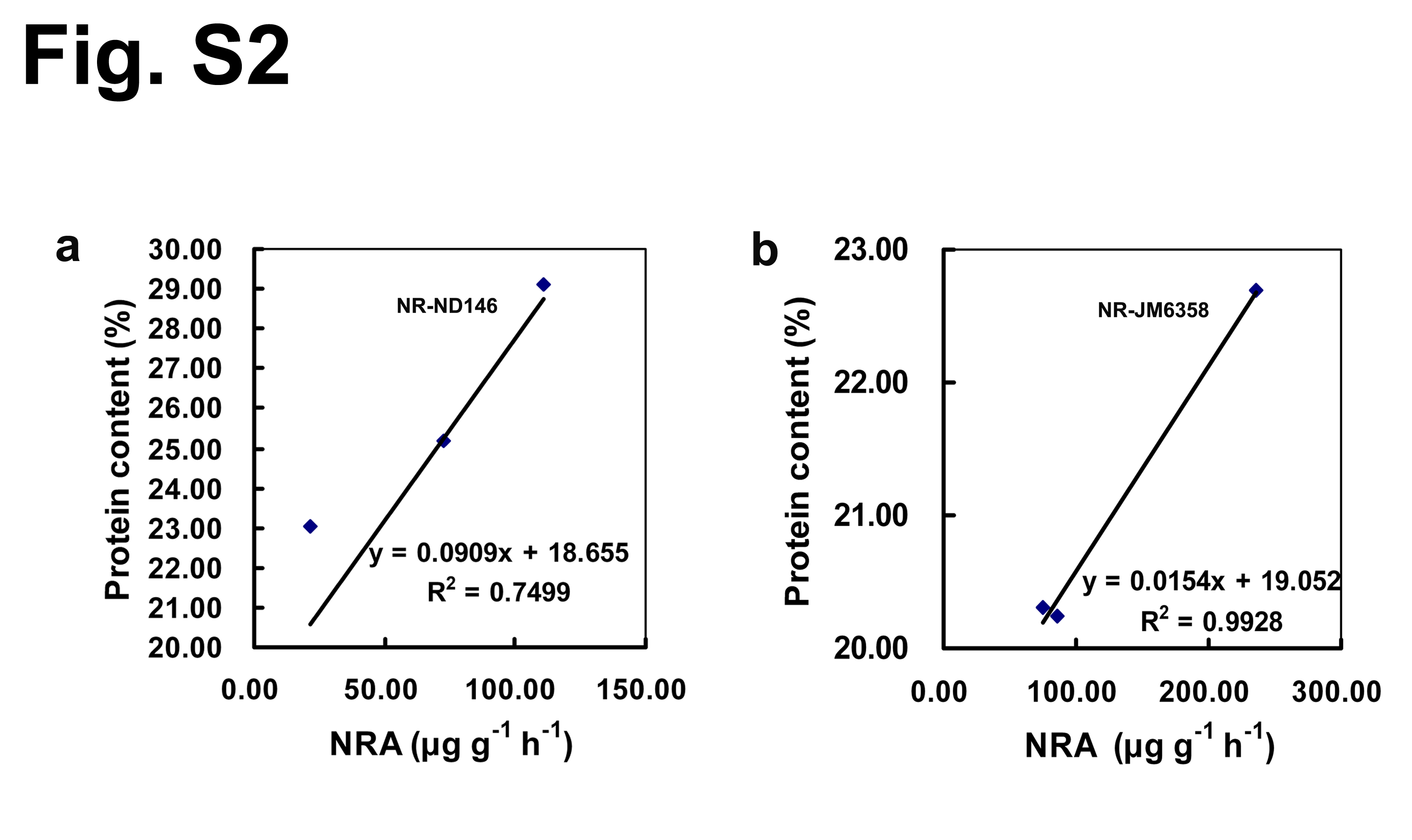

Supplement: Figure S2 — Relationship between T1 foliar NRA and T2 seed protein content of transgenic wheat. a: Leaf-sampled T1 offspring of NR-ND146. b: Leaf-sampled T1 offspring of NR-JM6358. (TIF) [file pone.0074678.s002.tif]

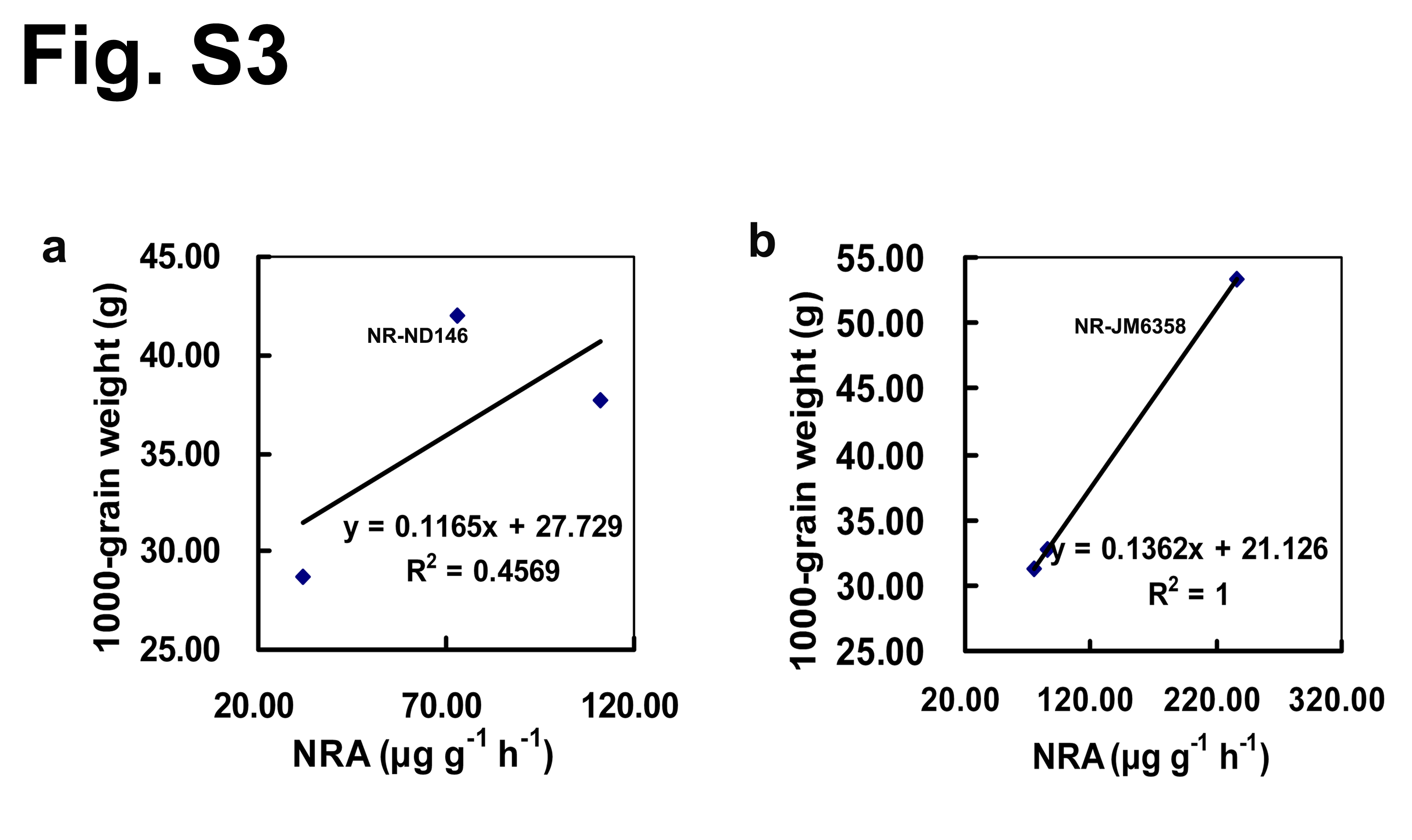

Supplement: Figure S3 — Relationship between T1 foliar NRA and T2 seed weight of transgenic wheat. a: Leaf-sampled T1 offspring of NR-ND146. b: Leaf-sampled T1 offspring of NR-JM6358. (TIF) [file pone.0074678.s003.tif]
